# Multitask Level-Based Learning Swarm Optimizer

**DOI:** 10.3390/biomimetics9110664

**Published:** 2024-11-01

**Authors:** Jiangtao Chen, Zijia Wang, Zheng Kou

**Affiliations:** 1 School Computer Science and Cyber Engineering, Guangzhou University, Guangzhou 510006, China; 2112306200@e.gzhu.edu.cn; 2 Institute of Computing Science and Technology, Guangzhou University, Guangzhou 510006, China

**Keywords:** evolutionary multitasking optimization (EMTO), particle swarm optimization (PSO), evolutionary operators

## Abstract

Evolutionary multitasking optimization (EMTO) is currently one of the hottest research topics that aims to utilize the correlation between tasks to optimize them simultaneously. Although many evolutionary multitask algorithms (EMTAs) based on traditional differential evolution (DE) and the genetic algorithm (GA) have been proposed, there are relatively few EMTAs based on particle swarm optimization (PSO). Compared with DE and GA, PSO has a faster convergence speed, especially during the later state of the evolutionary process. Therefore, this paper proposes a multitask level-based learning swarm optimizer (MTLLSO). In MTLLSO, multiple populations are maintained and each population corresponds to the optimization of one task separately using LLSO, leveraging high-level individuals with better fitness to guide the evolution of low-level individuals with worse fitness. When information transfer occurs, high-level individuals from a source population are used to guide the evolution of low-level individuals in the target population to facilitate the effectiveness of knowledge transfer. In this way, MTLLSO can obtain the satisfying balance between self-evolution and knowledge transfer. We have illustrated the effectiveness of MTLLSO on the CEC2017 benchmark, where MTLLSO significantly outperformed other compared algorithms in most problems.

## 1. Introduction

Evolutionary algorithms (EAs), including particle swarm optimization (PSO) algorithms [1,2,3], genetic algorithms (GAs) [4,5,6], ant colony optimization (ACO) algorithms [7,8], and differential evolution (DE) algorithms [9,10,11], have shown great effectiveness in solving many optimization problems, such as multi-objective [12,13,14], multimodal [15,16,17], and large-scale problems [18,19]. Due to its powerful capabilities and ease of implementation, EAs have been applied to many real-world problems, such as vehicle scheduling problems [20], protein structure prediction [21], cloud computing [22,23] and neural architecture search [24].

However, the traditional EAs were designed to optimize one task at a time, known as single-task optimization. When solving multiple tasks simultaneously, their performance often degrades and there exists much room for improvement. In reality, many tasks are often inherently correlated with each other. Therefore, the experience and knowledge gained from solving one problem often help in solving another problem. In this context, evolutionary multitasking optimization (EMTO), which simultaneously optimizes multiple tasks based on the knowledge transfer, has emerged as a new research topic [25]. It is important to note that although multi-objective optimization and multitask optimization may seem somewhat similar, they are fundamentally different. The goal of multi-objective optimization is to resolve conflicts between different objectives within a single task, balancing multiple objectives to obtain a set of solutions with different focuses, known as the Pareto front [26,27]. In contrast, multitask optimization simultaneously optimizes multiple tasks and leverages the similarity between tasks to transfer knowledge across them, thereby accelerating the algorithm’s search efficiency. The aim of multitask optimization is to find the global optimal solutions for multiple tasks simultaneously, without having to balance objectives across different tasks, as they do not conflict with each other.

In [25], the concept of multitask optimization was first proposed, and the multifactorial evolutionary algorithm (MFEA), the first multitask optimization algorithm, was introduced. Subsequently, many algorithms were proposed based on the framework of MFEA, called evolutionary multitask algorithms (EMTAs), including the GA-based multifactorial evolutionary algorithm with online transfer parameter estimation (MFEA-II) [28] and multitasking genetic algorithm (MTGA) [29], DE-based algorithms, multifactorial differential evolution (MFDE) [30], domain adaptation multitask optimization (DAMTO) [31], and block-level knowledge transfer for evolutionary multitask optimization (BLKT-DE) [32]. Among these multitask algorithms, almost all are based on GA or DE, whereas research on PSO-based EMTAs is relatively sparser. Compared with DE and GA, PSO has a faster convergence speed, especially during the later state of the evolutionary process. The traditional PSO algorithm achieves fast convergence by learning from its own historical best solution and the global historical best solution. However, due to solely relying on these two sources of information, the knowledge embedded in other particles within the swarm is not fully utilized, which may lead to premature convergence of the population. This issue also exists in the multifactorial particle swarm optimization (MFPSO) [30] algorithm, which only selects the global best solution from source task’s population to transfer. In addition, by always choosing the global best solution, the excellent information contained in other particles is lost, resulting in a decrease in the diversity of the transferred knowledge. Therefore, how to achieve efficient knowledge transfer is one of the key focuses in EMTO research.

To address the above-mentioned issues, we apply a level-based learning swarm optimizer (LLSO) [33] to multitask optimization, called the multitask level-based learning swarm optimizer (MTLLSO). In MTLLSO, multiple populations are maintained and each population corresponds to the optimization of one task using LLSO, where particles are categorized into different levels based on their fitness and each particle selects two different particles from higher levels for learning. The particles can significantly improve themselves by learning from the superior particles, leading to a full evolution. To enhance the diversity and effectiveness of knowledge transfer, we also applied the level-based learning strategy on the target population to learn from particles at different, higher levels in the source population, which promotes more diversified knowledge transfer between different populations, facilitating the effectiveness of knowledge transfer. In this way, MTLLSO can obtain the satisfying balance between self-evolution and knowledge transfer. Experimental results on the multitask benchmark CEC2017 [34] demonstrate the effectiveness of MTLLSO. The results indicate that MTLLSO significantly outperforms other multitask algorithms.

The remainder of this article is organized as follows: The related knowledge of LLSO and the related works of EMTO are given in Section 2. The MTLLSO is described in Section 3. The experimental studies are provided in Section 4. Section 5 concludes this article.

## 2. Preliminary

### 2.1. Particle Swarm Optimization

Particle swarm optimization (PSO) is a swarm intelligence algorithm inspired by the social behavior of birds flocking or fish schooling [1]. In PSO, the position of each particle in the swarm is a solution of problem. The search direction can be altered through learning from particles themselves and the leaders within the particle swarm. The particle swarm continuously changes its search direction in this way to find the optimal solution to the problem.

Assuming the use of PSO to solve a problem, where each position in the search space of the problem represents a particle, if the search space of the problem is *D*-dimensional and the particle swarm consists of *N* particles, then the position of the *i*th particle can be represented as *x_i_* = (*x_i_*_1_, *x_i_*_2_, …, *x_id_*). Each particle has a velocity *v_i_* = (*v_i_*_1_, *v_i_*_2_, …, *v_id_*), used to update its position, and a fitness value which evaluates the particle’s performance. The fitness is determined by the objective function being optimized. In the beginning, the swarm is usually initialized randomly across the entire search space of the problem. Subsequently, each particle in the swarm updates its velocity and position based on the best solution found by the swarm as a whole and its own personal best. The velocity and position updates for each particle in the particle swarm are mathematically defined as

*v_id_* =*ω* × *v_id_* + *c*_1_ × *r*_1_ × (*pbest_id_* − *x_id_*) + *c*_2_ × *r*_2_ × (*gbest_d_* − *x_id_*) (1)

*x_id_ = x_id_ + v_id_* (2)

where *pbest_id_* and *gbest_id_*, respectively, represent the best position of the *i*th particle and the global best position of the particle swarm. *id* indicates the *d*th dimension of the vector associated with the *i*th particle. *r*_1_ and *r*_2_ are two random numbers in [0, 1]. *ω* is the inertia coefficient, which can be a constant or linearly decayed. *c*_1_ and *c*_2_ are two positive constants used to control the influence of the two learning particles. After updating the velocity and position of the particles, they are re-evaluated; then, *pbest_id_* and *gbest_d_* are updated accordingly. The algorithm repeats this process until the termination condition is satisfied.

### 2.2. Level-Based Learning Swarm Optimizer

**Figure 1:**
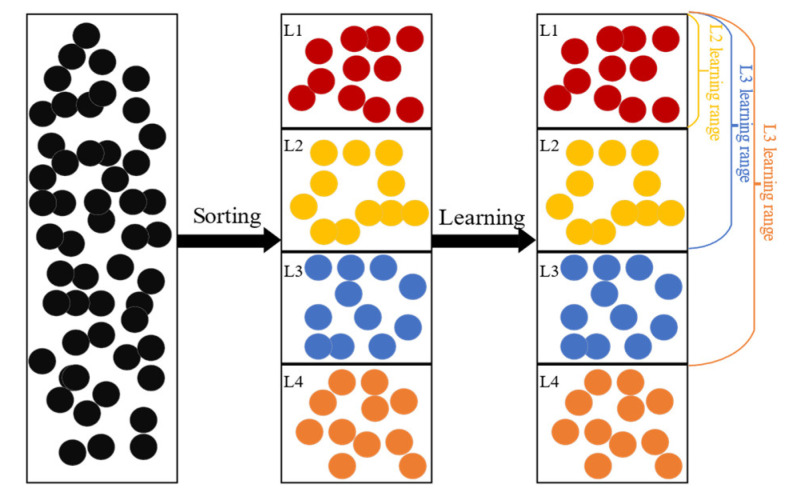
Structure of LLSO [33].

LLSO is a variant of PSO, so here we mainly discuss the differences between them [33], and the population structure of LLSO is shown in Figure 1.

In an *NP*-sized particle swarm, before each update, the particles in the swarm are sorted based on their fitness. Subsequently, they are divided into *L* levels based on their fitness, with each level containing [*NP*/*L*] particles. In this context, higher levels indicate better-performing particles, and the levels are arranged in ascending order. So, the highest level is *L*1. Unlike traditional PSO, in LLSO, particles learn from solutions of higher levels rather than *pbest* and *gbest*. Each level of particles can acquire different information, avoiding the loss of valuable solution information by learning from lower-level particles. It is important to note that particles at level *L*1 do not have higher levels to learn from, so they proceed directly to the next generation without updates, thus retaining valuable information for the next generation. The update formula for particles in LLSO is as follows:

*v_j,i_* = *r*_1_ × *v_j,i_* +*r*_2_ × (*x_k_*_1_ − *x_j,i_*) + *ϕ* × *r*_3_ × (*x_k_*_2_ − *x_j,i_*) (3)

*x_j,i_* = *x_j,i_* + *v_j,i_* (4)

Here, *v_j,i_* and *x_j,i_* represent the velocity vector and position vector of the *i*th particle in the *j*th level, respectively. *x_k_*_1_ and *x_k_*_2_ are randomly selected from particles at higher levels than the *j*th level, and *x_k_*_1_ is of a higher level than *x_k_*_2_, meaning that particle *x_k_*_1_ is better than particle *x_k_*_2_. Note that particles higher than level *L*2 only have particles from level *L*1 to learn from. Therefore, both learning particles for particles in level *L*2 are selected from particles in level *L*1. Thus, through this learning mechanism, the particle swarm maintains diversity and converges rapidly. *r*_1_, *r*_2_, and *r*_3_ are vectors of random numbers generated in [0, 1]. *ϕ* is a positive constant used to control the influence of poorer particles. LLSO repeats the above process until the termination conditions are met.

### 2.3. Multifactorial Evolutionary Algorithm

In [25], Gupta et al. introduced the concept of multitask optimization, which involves simultaneously optimizing multiple tasks. Meanwhile, MFEA, as the first multifactor optimization algorithm in the field of multitask optimization, was proposed in the same work. In MFEA, there are *N* tasks that need to be optimized simultaneously. Therefore, the solutions to each problem lie within a unified search space. The unified search space is a search space with a dimension of max(*D_i_*), where *D_i_* is the dimension of the task *T_i_*, and each dimension has a range of [0, 1]. During the evolution of the population, each individual’s solution is normalized to the range [0, 1] according to the upper and lower bounds of the corresponding optimization task, allowing tasks with different bounds and dimensional sizes to be optimized simultaneously in the same space. For optimization tasks with smaller dimensions, zero-padding is applied to individuals, with the number of zeros equal to the difference between the maximum dimension and the current task’s individual dimension. When evaluating individuals, decoding is performed using Equation (5) to map the individuals back to the original space, and the evaluation function is applied. Encoding is the inverse process of Equation (5).

*X_i_* = *L_i_* + (*U_i_* − *L_i_*) × *y_i_* (5)

Here, *L_i_* and *U_i_* are the lower and upper bounds of the *i*th variable of task *T_i_*. *y_i_* and *x_i_* represent the value of the *i*th variable of the individual in the unified search space and the original space, respectively.

To facilitate the evolution and comparison of individuals in a multitask environment, the following properties for each individual are defined:

- *Factorial cost*: In unconstrained optimization problems, for an individual *i* in the population, the factorial cost *f_i_* represents the fitness of individual *i* corresponding to task *T_i_*. If it is a constrained problem, a penalty term λ needs to be added.
- *Factorial rank:* The factorial rank *r_i_* represents the index of the factorial cost *f_i_* of individual *i* ranked in ascending order within task *T_i_*.
- *Scalar fitness:* The scalar fitness *φ_i_* represents the reciprocal of the optimal task rank of individual *i*. It is used to compare individuals across different tasks and is defined as *φ_i_* = 1/*min_j_*_∈{1, …,_ *_N_*_}_ $r_{i}^{j}$.
- *Skill factor:* The skill factor *τ_i_* of individual *i* denotes the task, which is the best performance among all tasks. *τ_i_* is given by *τ_i_* = *argmin*{$r_{i}^{j}$}, where *j*∈{1, …, *N*}.

MFEA is proposed based on the properties of individuals described above, and it achieves multitask optimization through two main modules: assortative mating and vertical transmission. In assortative mating, the optimization and knowledge transfer between individuals of the same task and different tasks are determined. The parameter *rmp* in assortative mating is set by the user, controlling the probability of offspring generation between individuals of different tasks, representing the extent of knowledge transfer. Vertical transmission is used to determine which parent’s skill factor is inherited by the offspring generated through assortative mating. Moreover, for each offspring individual, evaluation is performed only for the task corresponding to its skill factor. For more detailed information about the MFEA, readers can consult [25].

### 2.4. Related Work

EMTO is a new paradigm that leverages evolutionary algorithms (EAs) to solve multitask optimization problems, aiming to transfer knowledge between multiple optimization tasks to enhance the search capability of the algorithm. As the first EMTO algorithm proposed, MFEA has garnered significant attention. Many of the recently improved multitask algorithms are based on the MFEA multitask framework. Overall, the main research questions in EMTO are “how and what to transfer?” and “when to transfer?”.

Regarding the first question, researchers primarily focus on how to design effective knowledge transfer methods to ensure that knowledge is fully transferred and utilized between tasks. Existing transfer methods can mainly be divided into population-based knowledge transfer and individual-based knowledge transfer.

For individual-based knowledge transfer methods, researchers mainly focus on how to use individuals for knowledge transfer between different tasks. In MFEA, random selection of individuals for crossover is employed to achieve knowledge transfer. Besides that, many other methods have been proposed. For example, Zhou et al. [35] achieved adaptive selection of crossover operators by leveraging information from the multitask evolutionary process, enabling the algorithm to choose the appropriate crossover operator during knowledge transfer and improving the efficiency of knowledge transfer between individuals. Jiang et al. [32] further subdivided individuals into blocks and grouped them by similarity. By generating offspring within these clusters, knowledge transfer between misaligned dimensions is achieved, along with the reuse of knowledge across different dimensions within the same task.

For population-based knowledge transfer methods, researchers primarily explore how to transfer overall population information, i.e., transferring the knowledge of the source task population to individuals in the target task. For example, Feng et al. [36] constructed a denoising encoder between the source and target tasks, enabling the transfer of population information to individuals via the encoder. Li et al. [37] introduced the concept of meta-knowledge (knowledge that generates knowledge), transferring the center of the source task population to the center of the target task, thereby achieving the transfer of population meta-knowledge. Wu et al. [29] achieved population knowledge transfer by randomly transferring the bias from the source task to the target task in an unaligned manner.

Regarding the second question, “when to transfer”, many individual-based knowledge transfer algorithms use *rmp* to control the frequency of knowledge transfer. As a result, numerous researchers have proposed adaptive strategies for *rmp*. For instance, Li et al. [38] introduced a Q-table from reinforcement learning, treating the *rmp* setting as an action, enabling adaptive adjustment of *rmp* and enhancing the efficiency of knowledge transfer. Bali et al. [28] proposed an online *rmp* adaptive adjustment strategy, which evaluates the similarity between tasks to adjust *rmp* accordingly. In the study by Feng et al. [36], a fixed transfer interval is employed.

## 3. Proposed MTLLSO Algorithm

### 3.1. Motivation

After MFEA was proposed as the first EMTA, multitask optimization garnered widespread attention. In [25], MFEA employed GA operations for intra-population evolution and knowledge transfer between tasks. Subsequently, the multitask frameworks of the MFDE and MFPSO algorithms were proposed, utilizing DE and PSO algorithms, respectively [30]. Following this, an increasing number of multitask algorithms have been introduced, such as MFEA-II [28], MTGA [29], DAMTO [31], and BLKT-DE [32], all of which solely use GA or DE algorithms for intra-population evolution and knowledge transfer. Recently, some multi-evolution strategies like evolutionary multitasking via reinforcement learning (RLMFEA) [38] and MFEA with adaptive knowledge transfer (MFEA-AKT) [35] have also emerged. In RLMFEA, a population is initialized for each task, and the evolution and transfer strategies within the population are randomly determined as either DE or GA before each iteration. It can be observed that although PSO and DE are simultaneously applied in multitask evolutionary algorithms, research and applications using PSO algorithms have received relatively little attention. Additionally, we have discovered that the MFPSO algorithm, based on traditional PSO, only transfers the optimal solution from the source population during inter-population knowledge transfer. This method of transfer leads to overly singular knowledge transfer and overlooks the excellent information contained in other particles.

Based on the aforementioned issues, we incorporate LLSO into evolutionary multitask optimization. In MTLLSO, multiple populations first evolve separately using LLSO, utilizing high-level individuals to guide the evolution of low-level individuals. When information transfer occurs, high-level individuals from source populations are used to guide the evolution of low-level individuals in the target population. Thus, within populations, the advantages of LLSO are exploited for full evolution, while between populations, diverse knowledge transfer is achieved, aiding each other’s evolution.

### 3.2. Framework

The framework of MTLLSO is shown in Algorithm 1. Based on Algorithm 1, the MTLLSO is described as follows:

1. *P*_1_ and *P*_2_ are populations of two tasks randomly initialized in a unified search space.
2. Calculate the fitness of each particle for *P*_1_ and *P*_2_.
3. *P*_1_ and *P*_2_ are sorted in ascending order of fitness and divided into L groups respectively.
4. Generate offspring particles *OP*_1_ and *OP*_2_. Specifically, each particle of the target population determines whether to select particles from the source population or the target population based on the comparison result between *rand* and *rmp*. Subsequently, generate offspring particles using Equations (3) and (4). The aforementioned operations are repeated *NP* times for each population to obtain the corresponding offspring particle swarm. Then, evaluate their respective optimization tasks, i.e., the objective functions to be optimized. For more details, please refer to Algorithm 2.
5. Finally, concatenate the parent particle swarm with the corresponding offspring particle swarm based on the fitness to select the fittest *NP* particles to form the next particle swarm. The concatenation operation here refers to the concatenation operation of matrices.

**Algorithm 1:** MTLLSO  **Input**: the number of particles of two-task *NP*, random mating probability *rmp*, number of levels *L*, level size *LS*, control parameter *ϕ*, the maximum number of fitness evaluation *FES.*  /* Initialization */*fes* = 0 /* This variable counts the used fitness evaluations. */Randomly initialize *P*1 and *P*2 for two tasks respectively and evaluate the fitness values for P1 and P2;*fes* + = 2*NP;**While** *fes* < *FES* **do***P*1 and *P*2 are divided into L group;**For** *POP* = {*P*1, *P*2} **do** /* This variable the population evolving in the current iteration. */Generate offspring particles *OP* through Algorithm 2;Evaluate the generated offspring particles *OP*;*fes* + = (*NP* − *LS*);*POP* = *POP* ∪ *OP*;Sort *P*1 and *P*2 in ascending order of fitness;Select the fittest NP particles from *P*1 and *P*2 to form the next *P*1 and *P*2;**End while**  **Output**: The best solutions of *P*1 and *P*2

**Algorithm 2:** Level-based learning and knowledge transfer**  Input:** the source task particle swarm *P_s_*, the target task particle swarm *P_t_*, number of levels *L*, random mating probability *rmp*, level size *LS*, control parameter *ϕ*.  /* Initialization */*OP* = {}**For** *i* = {*L*, …, 2} **do****For** *j* = {1, …, *LS*} **do***x_new_* = *x_i,j_*;*v_new_* = *v_i,j_*;**If** *i* ! = 1 **then**Select two level from top (*i*−1) levels: *l*_1_, *l*_2_;**Else***l*_1_ = *l*_2_ = 1;**End If****If** *l*_1_ > *l*_2_ **then**Swap (*l*_1_, *l*_2_);**End If****If** *rand* < *rmp* **then**$\mathrm{Select}\;\mathrm{two}\;\mathrm{particles}\;\mathrm{from}\;l_{1},\;l_{2}\;\mathrm{respectively}\;\mathrm{in}\;P_{t}:\;x_{l_{1}}^{\prime },\;x_{l_{2}}^{\prime };$**Else**$\mathrm{Select}\;\mathrm{two}\;\mathrm{particles}\;\mathrm{from}\;l_{1},\;l_{2}\;\mathrm{respectively}\;\mathrm{in}\;P_{s}:\;x_{l_{1}},\;x_{l_{2}};$**End If**Update *v_new_* and *x_new_* according to Equations (3) and (4);*particle_new_* = {*v_new_*, *x_new_*_,_};*OP* = *OP* ∪ *particle_new_*;  **Output**: *OP*

### 3.3. Level-Based Knowledge Transfer

**Figure 2:**
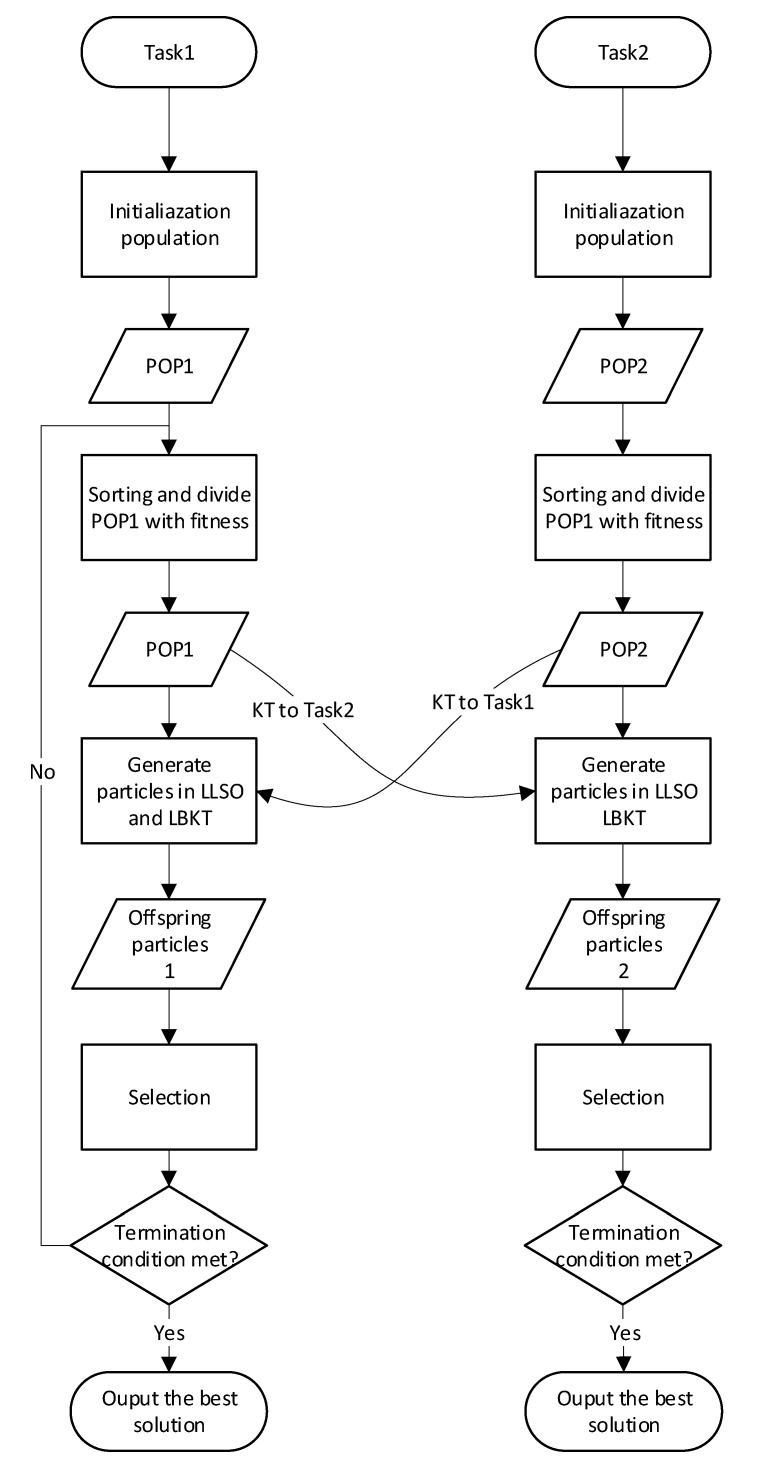
Workflow of the proposed MTLLSO.

In MFPSO, when conducting knowledge transfer between different tasks, the transferred knowledge all comes from the global best solution of the source population (Figure 2). Although the global best solution of the source population contains more information, transferring the same particle would lead to a loss of diversity in knowledge transfer. Therefore, to enhance the diversity of knowledge transfer, we need to select different advantageous particles from the source population as transfer targets. The updating strategy of LLSO is applicable not only within populations but also between populations. Thus, we can adopt the same strategy in the source population and select two different particles with higher ranks than its own for learning in the population of the source task, thereby achieving the strategy of knowledge transfer, called LBKT. Taking an *L*3-level particle update as an example, if the particle meets the knowledge transfer condition, one particle each from the *L*1-level and *L*2-level of the source population is selected as the learning target, denoted as x′*_k_*_1_ and x′*_k_*_2_. Then, Equation (4) is used for updating. Otherwise, the LLSO strategy is used for updating within the target population. For more details, please refer to Algorithm 2.

### 3.4. Complexity Analysis

The time complexity of evolutionary algorithms is typically analyzed under a given number of fitness evaluations, focusing only on the extra time per generation. The time spent on function evaluations is not considered, as it is unrelated to the algorithm itself. Compared to the original LLSO [33] algorithm, MTLLSO does not introduce any additional time overhead during knowledge transfer, so the time complexity of MTLLSO is the same as that of LLSO. Since the time complexity of PSO for updating particles in each generation is *O*(*NP* × *D*), and LLSO adds an operation to sort the population with a time complexity of *O*(*NP* log(*NP*) + *NP*), the time complexity of MTLLSO is *O*(*NP*(log(*NP*) + *D*)). In terms of space complexity, since the position and velocity of particles need to be stored, the space complexity is *O*(*NP* × *D*).

In conclusion, compared to the original LLSO, MTLLSO does not increase the time or space complexity.

## 4. Experiments and Result Analysis

To verify the performance of the MTLLSO algorithm, we conducted a test on the MTSO2017 benchmark. We compared MTLLSO with both single-evolutionary-operator algorithms and multi-evolutionary-operator algorithms. The single-evolutionary-operator algorithms include MFPSO, MFDE, MFPSO, MTGA, and evolutionary multitasking via explicit autoencoding (EMEA) [36], while the multi-evolutionary-operator algorithms include MFEA-AKT and RLMFEA. The comparison includes both earlier and more recent algorithms, which comprehensively demonstrates the effectiveness of MTLLSO. The details of MTLLSO can be observed from the code of MTLLSO, which is available at the following GitHub link: https://github.com/biggestfatboy/MTLLSO (accessed on 18 Oct 2024).

### 4.1. Experimental Setting

The experimental parameters of MTLLSO and other algorithms are set as follows:

The FES is set the same as the requirement of the CEC2017MTSO benchmark for fairness, with the same for the other compared algorithms. The other parameters are set as follows:

(1) *NP* is set as 100, which is a common setting in MTO [28,29,30,31,32].
(2) *L* and *ϕ* are set as 4 and 0.4, respectively, which are the same as the original LLSO paper.
(3) *rmp* is set as 0.1, which is the optimal parameter based on our experimental comparisons in Section 4.4.

For fairness, the parameters of other algorithms are kept the same as in their original papers.

Table 1 presents the test results of MTLLSO and other algorithms on the CEC2017 MTSO benchmarks. The data in Table 1 represent the average fitness of the best solution obtained by the algorithm after 30 independent runs on the corresponding problem. The best results are highlighted in bold. Additionally, the symbols “+/≈/-” indicate whether the results of the MTLLSO are better than, equal to, or worse than those of the comparison algorithms under the Wilcoxon rank-sum test.

### 4.2. The Result of CEC2017MTSO Benchmarks

The CEC2017MTSO benchmarks are artificially designed test problems used to evaluate the performance of algorithms in solving multitask optimization problems. CEC2017MTSO benchmarks mainly consist of nine multitask optimization problems, each of which contains two minimization tasks that need to be optimized simultaneously. Based on the intersection of the optimal solutions of the two tasks in the unified search space, the problems are categorized into complete intersection (CI), partial intersection (PI), and no intersection (NI). Furthermore, based on the synergy between tasks, the problems are subdivided into high similarity (HS), medium similarity (MS), and low similarity (LS). For example, the CIHS problem involves simultaneously optimizing two tasks that share the same global optimum and exhibit high similarity. The specific functions for the two tasks to be optimized are the rotated Rastrigin and rotated Griewank functions. In the CEC2017MTSO benchmarks, for each multitask optimization problem, the maximum number of FES is 100,000. Therefore, when using this test set, for a fair comparison, the same FES as the CEC2017MTSO benchmarks is adopted. For more details on other functions, please refer to http://www.bdsc.site/websites/MTO/index.html#contact or http://www.bdsc.site/websites/MTO/index.html. (accessed on 18 October 2024).

According to Table 1, we can observe that on the CEC2017 benchmark, MTLLSO outperforms or performs similarly to MFPSO, EMEA, MFDE, MFEA, MTGA, MFEA-AKT, and RLMFEA in eight, eight, six, seven, six, seven, and six problems, respectively. Conversely, the number of instances where MTLLSO exhibits poorer performance on individual tasks is one, one, three, three, four, three, and five. Compared to MFPSO, MTLLSO has made significant progress, with MTLLSO outperforming MFPSO comprehensively except for CILS-T1. The main difference between the two lies in their particle updating methods. Within the population, MTLLSO adopts the LLSO update strategy, using dominant particles within the current population as learning examples, enhancing population diversity, expanding the population’s search range. During knowledge transfer between populations, the LLSO update strategy enables comprehensive transfer of knowledge from one population to another, enhancing the diversity of knowledge transfer. Moreover, compared to recently proposed algorithms, MTLLSO also demonstrates significant advantages, proving the effectiveness of MTLLSO.

**Figure 3:**
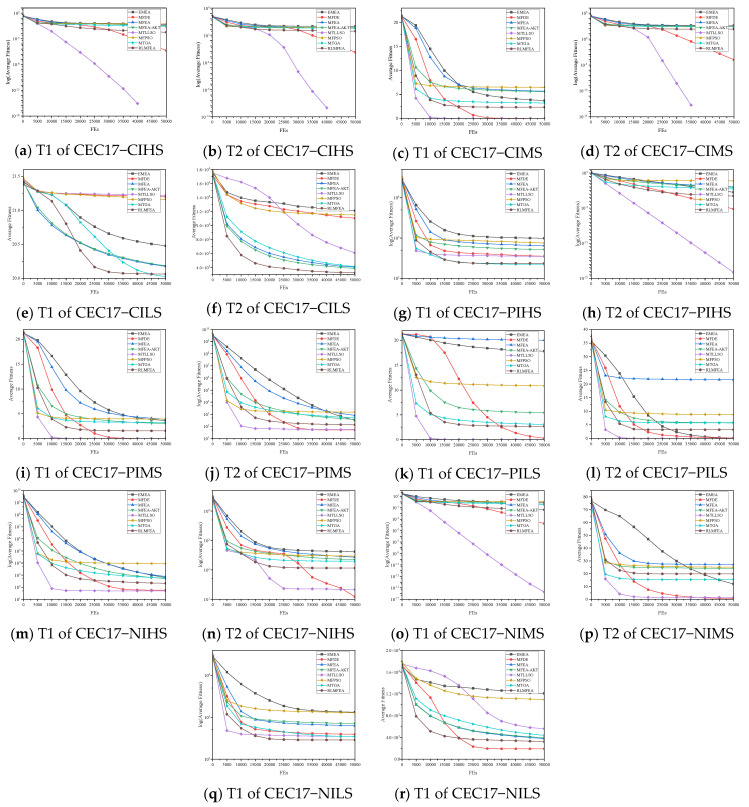
Convergence curves of the average fitness on CEC2017MTSO over 30 independent runs.

To further explore the performance of MTLLSO, Figure 3 depicts the average convergence characteristics of MTLLSO, MFPSO, EMEA, MFDE, MFEA, MTGA, MFEA-AKT, and RLMFEA. Please note that we are optimizing minimization problems, so the lower the fitness, the better. As shown in Figure 3, except for the subpar convergence performance on CILS and NILS-T2, MTLLSO demonstrates significantly better convergence speed compared to or similar to other algorithms on the remaining problems. Although MTLLSO did not achieve the best results on PIHS-T2, NIHS-T2, NIMS-T2, and NILS-T1, it still maintains a strong competitive advantage in convergence speed. MTLLSO achieves the best convergence speed and performance on problems such as CIHS, CIMS, PIMS, and PILS. Moreover, observing Figure 3, we notice that MTLLSO exhibits rapid convergence in the early stages of evolution in PIHS-T1 but becomes trapped in local optima in the later stages. Conversely, in PIHS-T2, MTLLSO almost continuously converges without stagnation. This divergence might stem from transferring knowledge from the PIHS-T2 population to the PIHS-T1 population, accelerating the evolution of the latter but also leading to premature convergence. In the CILS problem, algorithms utilizing GA evolutionary operators significantly outperform those using DE or PSO operators, indicating GA operators’ suitability for such problems. On the other hand, in the NILS problem, MTLLSO’s convergence speed and performance on NILS-T2 are much lower than NILS-T1, with algorithms using DE or GA operators yielding results comparable to or better than MTLLSO. This suggests that DE and GA operators might be more suitable for solving NILS. Additionally, as shown in Figure 3, except for CILS-T1, MTLLSO outperforms MFPSO on all other problems, demonstrating that MTLLSO is more suitable for multitask optimization than MFPSO.

Different evolutionary operators demonstrate distinct advantages in specific problem-solving scenarios, while multi-operator algorithms exhibit significant benefits across most problems. Therefore, although MTLLSO performs well on most problems, combining it with other evolutionary operators can enhance its performance in certain cases.

### 4.3. Time Comparison Analysis

In this section, we compare and analyze the execution time of MTLLSO with other EMTO algorithms. Since the experimental data of MFEA-AKT were obtained using Matlab source code, while the other algorithms were reproduced using Python based on the original papers, comparing the execution times across different programming languages seems unfair. Therefore, we only compare MTLLSO with EMEA, MFDE, MFEA, MFPSO, MTGA, and RLMFEA. The comparison results of the average execution time of MTLLSO and other algorithms on the CEC2017 MTSO benchmarks are shown in Table 2. From Table 2, it can be seen that, except for MFPSO, our algorithm takes less time than other algorithms on the majority of problems. Although our algorithm takes more time than MFPSO, Table 1 shows that the quality of the solution we obtain is better. In other words, the increased time cost of MTLLSO can be compensated by better solution accuracy.

### 4.4. Parameter Sensitivity

We mainly focus on the study of the parameter setting sensitivity of *rmp*. We primarily study the sensitivity of the *rmp* parameter setting. The value of *rmp* controls the frequency of knowledge transfer between tasks in each generation. We conducted experiments on the CEC2017 MTSO benchmarks, setting the *rmp* parameter in MTLLSO as *rmp* ∈ {0.1, 0.2, 0.3, 0.4, 0.5, 0.6, 0.7, 0.8, 0.9}, with other parameters remaining the same. The experimental results of the average fitness of the best solution over 30 independent runs are shown in Table 3.

From Table 3, it can be seen that as *rmp* increases, the performance of the algorithm gradually decreases. When *rmp* is set to 0.1, except for the task PILS-T2 where it performs worse than MTLLSO with *rmp* set to 0.2, it outperforms or is comparable to other *rmp* settings on all other tasks. The best results are observed when *rmp* is set to 0.1. Therefore, we chose 0.1 as the *rmp* of MTLLSO.

## 5. Conclusions

To further investigate the effectiveness of particle swarm optimization in EMTO, we apply LLSO to multitask optimization and propose the multitask level-based learning swarm optimizer (MTLLSO). Compared to other multitask algorithms, MTLLSO achieves more effective knowledge transfer during knowledge migration, enhancing the search capability of algorithm. During intra-swarm evolution, the LLSO update strategy is adopted, where the high-level individuals with better fitness guide the evolution of low-level individuals with worse fitness. When information transfer occurs, high-level individuals from the source population are used to guide the evolution of low-level individuals in the target population to facilitate the effectiveness of knowledge transfer.

In this way, MTLLSO can obtain the satisfying balance between self-evolution and knowledge transfer. Experimental results on the CEC2017 multitask benchmarks demonstrate the effectiveness of the MTLLSO.

Currently, there is not yet a large-sized MTO benchmark available. In future work, we will further investigate and enhance the performance of MTLLSO on large-scale MTO. Additionally, we aim to investigate the use of machine learning methods to achieve knowledge transfer in multitask optimization and hope to combine MTLLSO with other evolutionary operators to further explore their capabilities in the multitask optimization domain.

## Figures and Tables

**Table 1:** The CEC17 experimental results of MTLLSO and other EMTO algorithms.

Question		MTLLSO	MFPSO	EMEA	MFDE	MFEA	MTGA	MFEA-AKT	RLMFEA
CEC17-CIHS	T1	**0.00e+00**	8.17e−01 (+)	5.66e−01 (+)	5.46e−06 (+)	3.80e−01 (+)	2.72e−01 (+)	3.42e−01 (+)	1.89e−02 (+)
	T2	**0.00e+00**	2.54e+02 (+)	4.13e+02 (+)	7.24e−03 (+)	2.04e+02 (+)	2.05e+02 (+)	1.86e+02 (+)	5.42e+01 (+)
CEC17-CIMS	T1	**5.91e−13**	6.47e+00 (+)	3.70e+00 (+)	3.95e−04 (+)	5.67e+00 (+)	3.21e+00 (+)	5.57e+00 (+)	2.32e+00 (+)
	T2	**0.00e+00**	2.86e+02 (+)	4.14e+02 (+)	1.02e−04 (+)	2.71e+02 (+)	2.36e+02 (+)	2.54e+02 (+)	8.61e+01 (+)
CEC17-CILS	T1	2.12e+01	2.12e+01 (-)	2.05e+01 (-)	2.12e+01 (≈)	2.02e+01 (-)	**2.00e+01 (-)**	2.02e+01 (-)	2.01e+01 (-)
	T2	6.08e+03	1.15e+04 (+)	1.21e+04 (+)	1.10e+04 (+)	4.04e+03 (-)	4.11e+03 (-)	3.85e+03 (-)	**3.24e+03 (-)**
CEC17-PIHS	T1	3.50e+02	7.54e+02 (+)	9.92e+02 (+)	3.54e+02 (+)	6.50e+02 (+)	**2.24e+02 (-)**	5.17e+02 (+)	2.37e+02 (-)
	T2	**5.03e−24**	6.28e+02 (+)	3.43e−01 (+)	5.91e−06 (+)	1.18e+01 (+)	3.09e+00 (+)	9.07e+00 (+)	2.96e−02 (+)
CEC17-PIMS	T1	**7.42e−13**	3.93e+00 (+)	3.63e+00 (+)	1.26e−03 (+)	3.85e+00 (+)	3.28e+00 (+)	3.02e+00 (+)	1.54e+00 (+)
	T2	5.43e+01	1.52e+03 (+)	3.19e+02 (+)	**5.05e+01 (≈)**	8.16e+02 (+)	5.14e+02 (+)	3.74e+02 (+)	1.35e+02 (+)
CEC17-PILS	T1	**8.43e−13**	1.09e+01 (+)	1.78e+01 (+)	2.58e−01 (+)	2.00e+01 (+)	3.06e+00 (+)	5.41e+00 (+)	2.68e+00 (+)
	T2	**8.80e−03**	8.76e+00 (+)	1.71e−01 (+)	1.53e−01 (+)	2.16e+01 (+)	5.72e+00 (+)	5.81e+00 (+)	3.21e+00 (+)
CEC17-NIHS	T1	**4.93e+01**	9.02e+03 (+)	6.37e+02 (+)	5.63e+01 (+)	7.68e+02 (+)	5.68e+02 (+)	5.18e+02 (+)	2.12e+02 (+)
	T2	2.14e+01	2.93e+02 (+)	4.16e+02 (+)	**1.22e+01 (-)**	2.71e+02 (+)	1.98e+02 (+)	2.20e+02 (+)	1.16e+02 (+)
CEC17-NIMS	T1	**1.92e−16**	1.16e+00 (+)	7.31e−01 (+)	1.72e−04 (+)	4.17e−01 (+)	3.79e−01 (+)	4.13e−01 (+)	4.27e−02 (+)
	T2	1.46e+00	2.51e+01 (+)	1.20e+01 (+)	**4.79e−01 (-)**	2.73e+01 (+)	1.54e+01 (+)	2.42e+01 (+)	1.99e+01 (+)
CEC17-NILS	T1	3.46e+02	1.28e+03 (+)	1.32e+03 (+)	3.92e+02 (+)	6.27e+02 (+)	3.44e+02 (≈)	7.03e+02 (+)	2.89e+02 (-)
	T2	5.58e+03	1.09e+04 (+)	1.21e+04 (+)	**1.91e+03 (-)**	3.77e+03 (-)	4.36e+03 (-)	3.90e+03 (-)	3.23e+03 (-)
Number of +/≈/-			17/0/1	17/0/1	13/2/3	15/0/3	13/1/4	15/0/3	13/0/5

**Table 2:** Computation times of MTLLSO and other EMTO algorithms.

Task	MTLLSO	EMEA	MFDE	MFEA	MFPSO	MTGA	RLMFEA
CIHS	3.68s	7.59s (+)	4.38s (+)	8.39s (+)	3.85s (+)	9.41s (+)	7.93s (+)
CIMS	3.97s	6.91s (+)	4.75s (+)	8.72s (+)	4.27s (+)	9.52s (+)	8.34s (+)
CILS	3.75s	6.34s (+)	4.70s (+)	8.08s (+)	3.61s (-)	8.91s (+)	8.30s (+)
PIHS	3.34s	6.03s (+)	3.86s (+)	8.16s (+)	3.11s (-)	8.82s (+)	8.15s (+)
PIMS	3.69s	6.44s (+)	4.47s (+)	8.64s (+)	3.62s (-)	9.55s (+)	8.26s (+)
PILS	18.99s	18.27s (-)	20.13s (+)	24.20s (+)	18.95s (≈)	24.88s (+)	21.59s (+)
NIHS	3.73s	6.09s (+)	4.40s (+)	8.44s (+)	3.28s (-)	8.69s (+)	7.87s (+)
NIMS	34.95s	31.45s (-)	35.17s (≈)	40.79s (+)	35.98s (+)	41.46s (+)	34.43s (-)
NILS	3.40s	6.02s (+)	4.46s (+)	9.25s (+)	3.23s (-)	7.89s (+)	9.16s (+)
Number of +/≈/-		7/0/2	8/1/0	9/0/0	3/1/5	9/0/0	8/0/1

**Table 3:** The CEC17 experimental results of MTLLSO with different *rmp* parameter settings.

Question		MTLLSO								
		*rmp* = 0.1	*rmp* = 0.2	*rmp* = 0.3	*rmp* = 0.4	*rmp* = 0.5	*rmp* = 0.6	*rmp* = 0.7	*rmp* = 0.8	*rmp* = 0.9
CEC17-CIHS	T1	**2.47e−04**	**0.00e+00 (≈)**	**0.00e+00 (≈)**	**0.00e+00 (≈)**	**0.00e+00 (≈)**	**0.00e+00 (≈)**	**0.00e+00 (≈)**	**0.00e+00 (≈)**	**0.00e+00 (≈)**
	T2	**1.05e+01**	**0.00e+00 (≈)**	**0.00e+00 (≈)**	**0.00e+00 (≈)**	**0.00e+00 (≈)**	**0.00e+00 (≈)**	**0.00e+00 (≈)**	**2.46e−14 (+)**	1.19e−13 (+)
CEC17-CIMS	T1	**6.11e−13**	1.52e−12 (+)	4.13e−12 (+)	1.55e−10 (+)	3.52e−11 (+)	9.84e−11 (+)	3.35e−10 (+)	1.03e−09 (+)	2.95e−09 (+)
	T2	**0.00e+00**	**0.00e+00 (≈)**	**0.00e+00 (≈)**	**3.32e−02 (≈)**	**0.00e+00 (≈)**	**0.00e+00 (≈)**	**0.00e+00 (≈)**	**0.00e+00 (≈)**	**0.00e+00 (≈)**
CEC17-CILS	T1	**2.12e+01**	**2.12e+01 (≈)**	**2.12e+01 (≈)**	**2.12e+01 (≈)**	**2.12e+01 (≈)**	**2.12e+01 (≈)**	2.12e+01 (+)	**2.12e+01 (≈)**	**2.12e+01 (≈)**
	T2	**5.86e+03**	**8.57e+03 (≈)**	1.15e+04 (+)	1.33e+04 (+)	1.52e+04 (+)	1.59e+04 (+)	1.62e+04 (+)	1.63e+04 (+)	1.64e+04 (+)
CEC17-PIHS	T1	**3.51e+02**	**3.54e+02 (≈)**	3.47e+02 (≈)	3.59e+02 (+)	3.63e+02 (+)	3.71e+02 (+)	3.80e+02 (+)	3.87e+02 (+)	4.26e+02 (+)
	T2	**5.70e−24**	1.10e−21 (+)	2.99e−19 (+)	1.24e−16 (+)	8.82e−14 (+)	5.44e−11 (+)	1.04e−07 (+)	1.75e−04 (+)	6.60e−01 (+)
CEC17-PIMS	T1	**8.45e−13**	9.38e−12 (+)	1.37e−10 (+)	1.72e−09 (+)	2.97e−08 (+)	3.68e−07 (+)	6.34e−06 (+)	1.53e−04 (+)	3.89e−03 (+)
	T2	**5.43e+01**	6.58e+01 (+)	7.38e+01 (+)	7.62e+01 (+)	8.05e+01 (+)	8.45e+01 (+)	8.47e+01 (+)	8.52e+01 (+)	8.68e+01 (+)
CEC17-PILS	T1	**9.01e−13**	1.21e−11 (+)	2.23e−10 (+)	4.87e−09 (+)	3.08e−05 (+)	1.03e−02 (+)	9.91e−03 (+)	2.57e−03 (+)	1.04e−03 (+)
	T2	2.70e−02	**1.03e−02 (-)**	1.18e−02 (≈)	2.32e−02 (≈)	2.52e−02 (≈)	4.80e−02 (≈)	5.21e−02 (+)	3.19e−02 (+)	8.48e−03 (+)
CEC17-NIHS	T1	**4.53e+01**	**4.54e+01 (≈)**	**4.51e+01 (≈)**	**4.53e+01 (≈)**	**4.52e+01 (≈)**	**4.53e+01 (≈)**	4.57e+01 (+)	4.61e+01 (+)	4.71e+01 (+)
	T2	**3.32e−02**	**0.00e+00 (≈)**	**2.98e−01 (≈)**	**9.47e−15 (≈)**	3.32e−02 (+)	6.63e−02 (+)	2.85e−08 (+)	7.52e−06 (+)	1.83e−03 (+)
CEC17-NIMS	T1	**2.47e−04**	3.87e−13 (+)	4.93e−04 (+)	4.93e−04 (+)	4.93e−04 (+)	2.47e−04 (+)	1.07e−03 (+)	1.16e−03 (+)	4.91e−02 (+)
	T2	**1.15e+00**	**1.30e+00 (≈)**	**1.39e+00 (≈)**	1.65e+00 (+)	2.06e+00 (+)	2.30e+00 (+)	3.03e+00 (+)	4.03e+00 (+)	7.66e+00 (+)
CEC17-NILS	T1	**3.46e+02**	**3.55e+02 (≈)**	3.57e+02 (+)	3.62e+02 (+)	3.64e+02 (+)	3.71e+02 (+)	3.82e+02 (+)	4.01e+02 (+)	4.36e+02 (+)
	T2	**5.99e+03**	**5.73e+03 (≈)**	**6.26e+03 (≈)**	9.01e+03 (+)	1.17e+04 (+)	1.39e+04 (+)	1.57e+04 (+)	1.66e+04 (+)	1.70e+04 (+)
Number of +/≈/-			6/11/1	8/10/0	11/7/0	12/6/0	12/6/0	15/3/0	15/3/0	15/3/0

## Data Availability

The original contributions presented in the study are included in the article. Further inquiries can be directed to the corresponding authors first.

## Nomenclature

**Abbreviations**	**Full Name**
EMTO	Evolutionary multitasking optimization
EMTAs	Evolutionary multitask algorithms
DE	Differential evolution
GA	Genetic algorithm
PSO	Particle swarm optimization
ACO	Ant colony optimization
MTLLSO	Multitask level-based learning swarm optimizer
MFEA	Multifactorial evolutionary algorithm
MFEA-II	Multifactorial evolutionary algorithm with online transfer parameter estimation
MTGA	Multitasking genetic algorithm
MFDE	Multifactorial differential evolution
DAMTO	Domain adaptation multitask optimization
BLKT-DE	Block-level knowledge transfer for evolutionary multitask optimization
MFPSO	Multifactorial particle swarm optimization
LLSO	Level-based learning swarm optimizer
RLMFEA	Evolutionary multitasking via reinforcement learning
MFEA-AKT	MFEA with adaptive knowledge transfer
LBKT	Level-based knowledge transfer
EMEA	Evolutionary multitasking via explicit autoencoding
CI	Complete intersection
PI	Partial intersection
NI	No intersection
HS	High similarity
MS	Medium similarity
LS	Low similarity
